# Type, Number or Both? A Population-Based Matched Case-Control Study on the Risk of Fall Injuries among Older People and Number of Medications beyond Fall-Inducing Drugs

**DOI:** 10.1371/journal.pone.0123390

**Published:** 2015-03-27

**Authors:** Lucie Laflamme, Joel Monárrez-Espino, Kristina Johnell, Berty Elling, Jette Möller

**Affiliations:** 1 Karolinska Institutet, Department of Public Health Sciences, Stockholm, Sweden; 2 Aging Research Center, Karolinska Institutet and Stockholm University, Stockholm, Sweden; Heinrich-Heine University, Faculty of Medicine, GERMANY

## Abstract

**Objectives:**

Drug use is a modifiable risk factor for fall-related injuries in older people. Whereas the injurious effect of polypharmacy is established, that of low numbers of medications has not been fully ascertained. Neither do we know whether it is the number per se or the type of medications that actually matters. We assessed this question for fall injuries leading to hospitalization.

**Design:**

National register-based, population-based, matched case-control study.

**Setting:**

Community dwellers aged 65+ years living in Sweden between March 2006 and December 2009.

**Methods:**

Cases (n = 64,399) were identified in the national inpatient register and four controls per case were randomly matched by gender, date of birth and residential area. The association between number of prescribed medications, assessed through linkage with the Swedish prescribed drug register, and the risk of injurious falls was estimated with odds ratios with 95% confidence intervals using conditional logistic regression, adjusted for demographic and health status.

**Results:**

The number of medications was associated with an increased risk of fall injury in a dose-response fashion, even after adjustment for marital status, comorbidity and number of fall-risk-inducing drugs (FRIDs). Using ten or more medications was associated with an almost two-fold higher risk (adjusted OR: 1.76, 95% CI: 1.66 to 1.88). When stratified by use (or not) of at least one FRID, the association weakened slightly among both non-users (adjusted OR: 1.50, 95% CI: 1.34 to 1.67) and users (adjusted OR: 1.67, 95% CI: 1.58 to 1.77).

**Conclusion:**

In older people, not only large but also small numbers of medications may affect the risk for them to sustain injurious falls. Although the mechanisms lying behind this are complex, the finding challenges the prevention strategies targeting either specific types of medications (FRIDs) or high numbers of them.

## Introduction

Recent estimates reveal that non-fatal health outcomes are on the rise in nearly all parts of the world and in all age groups, including elderly people [1]. Injuries, in particular those resulting from falls, such as femur fracture, are dangerous and incapacitating for this group [2–5]. They usually require hospitalization and costly interventions and cause many of the functional limitations that lead to the need for long-term care, including admissions to nursing homes [3]. Globally, fall-related injuries among older people are unevenly distributed across countries and they occur to a greater extent in high-income countries, not least in Europe [6]. Sweden, where the current study was conducted, has not only one of the oldest populations in the world, but also one of the highest incidences of fall-related injuries among older people [7]. For instance, it has been estimated that the life-time risk of a hip fracture at the age of 50 years is 28.5% among women and 13.1% among men [8] with 75% of the victims being women aged 81 years on average [9].

Medication use is a modifiable risk factor for falls and fall-related injuries and there is considerable research interest in this association among older people [5, 10]. Fall-risk-inducing drugs (FRIDs) are many, and have been summarized in several recent reviews and meta-analyses [5, 11–14]. The use of sedatives and hypnotics, such as the benzodiazepines as well as anti-depressants, has been shown to have the strongest associations with falling and fall-related injuries [14]. Other drugs with documented associations are antipsychotics, antiepileptics, antiparkinsonian drugs, cardiac medications, opioids and urological spasmolytics [5, 11–14].

There is also a growing body of knowledge, albeit from relatively small studies, on the effect of number—rather than the specific types—of medications on fall injuries among older people. Polypharmacy alone has been associated with an increased risk of falls leading to hospital visit or hospitalization [15–18], of falls sustained during a hospital stay [19], and of negative health consequences among trauma patients like complications, lower functional outcomes and longer hospital and intensive care stays [20]. There is a suggestion, but insufficient evidence, that polypharmacy, rather than comorbidities, determine those associations [17, 19]. A study on home-related injuries in an adult population also suggests that the combination of relatively few medications may affect the risk of injury [21] but, to our knowledge, this has not yet been investigated in older people.

We aim to fill this knowledge gap by studying the association between an increasing number of medications and fall-related injuries among older people considering a national population-based setting, taking into consideration the eventual role played by the consumption of FRIDs.

## Methods

### Study design

We conducted a matched case-control study using several population-based registers linked through the unique personal identification number assigned to all residents in Sweden. From a national cohort of 6 981 010 individuals born before 1959, and domiciled in Sweden at some point from 1973 onwards (identified using the Total Population Register), we selected cases 65 years and older hospitalized due to a fall injury during the period between 1 March 2006 and 31 December 2009.

### Selection of cases and controls

Cases were identified using the Swedish National Patient Register. Discharge diagnoses, based on the International Classification of Diseases, Tenth Revision (ICD 10), were used for the definition of a fall injury; the external cause and morbidity and mortality codes classified as W00—W19. Only the first episode of hospitalization during the study period was considered and planned inpatient care with its corresponding diagnostic codes was excluded.

Eligible as controls were those in the national cohort not hospitalized due to a fall injury during the study period. Controls, four per case, were randomly selected and matched to the cases by sex, date of birth and area of residence. After matching to a case, the controls were censored so that they could not be assigned to another case. The day of hospital admission, and corresponding date for the controls, was used as the index date. A total of 64 399 cases (22 190 males and 42 209 females) and 257 596 controls were identified—including four controls per case—adding up to a total of 321 995 persons included in this study, with the age distribution; 65–69, 70–79, 80–89, and 90+ years (respectively 4.3%, 25.1%, 51.5% and 19.1% of the study group).

### Exposure

The Swedish Prescribed Drug Register, where all dispensed prescriptions have been registered for all residents in Sweden since July 2005, was used to assess the dispensation date and the type of medication based on the Anatomical Therapeutic Chemical (ATC), based on the full five-digit ATC code classification [22]. As for the exposure period, we used 1–30 days prior to the index date as the association we studied—number of medications and the occurrence of injurious falls—required that focus was placed on those medications used in a time period close to the injury. The total number of unique dispensed drugs during the 30 day period was used to determine the number of medications, excluding the dispensation day assuming that medication was not taken on that date, but one day after. Number of medications was coded into 0, 1, 2, 3, 4, 5, 6, 7, 8, 9 and 10 or more different medications.

### Covariates

The individuals’ age and sex were extracted from the Total Population Register while area of residence and marital status were extracted from the Longitudinal Integrated databases on the Insurance and Labor Market (LISA). The most recently registered marital status, area of residence prior to and age at the index date was used.

FRIDs were assessed based on the same information as number of medications and defined following the classification used by the Swedish National Board of Health and Welfare [23]. The list of FRIDs, with their corresponding ATC codes, included: vasodilators used in cardiac diseases (C01D), antihypertensive drugs (C02), diuretics (C03), beta blocking agents (C07), calcium channel blockers (C08), agents acting on the renin-angiotensin system (C09), alpha-adrenoreceptor antagonists (for benign prostatic hypertrophy) (G04CA), opioids (N02A), dopaminergic agents (anti-Parkinson drugs, N04B), antipsychotics excl. lithium (N05A excl. N05AN), anxiolytics (N05B), hypnotics and sedatives (N05C) and antidepressants (N06A). Number of FRIDs during the 30-day period prior to index date was computed and classified as 0, 1, 2, 3, 4 and 5 or more.

Data were extracted from the National Patient Register to calculate comorbidity according to the updated Charlson comorbidity weighted index by QUAN, herein referred to CCI, which shows the number and seriousness of comorbid diseases. The index contains 17 categories of comorbidities defined based on ICD-10 diagnoses and each category has an associated weight from 1 to 6 [24, 25]. We used history of hospital admissions based on the main discharge diagnoses within 1 and 3 years prior to the index date. However, since there was practically no difference in the scores, we adjusted for comorbidity status within the previous year to get closer to the index date. We categorized CCI into the following 4 groups: 0, 1–2, 3–4 and 5 or more, with a higher score indicating more serious comorbidity which was then used to take confounding by indication into account. Due to the limited availability of health status measures in the registers we used the number of hospitalisation days as an alternative measure of comorbidity. The total number of days in hospital during the year prior to index date was calculated and categorized into the following groups: 0, 1, 3–4, 4–7, 8–15, 15–30 and more than 30 days.

### Statistical analyses

Frequencies were used to describe the age distribution of fall injured and controls (65–69, 70–79, 80–89 and 90+ years), sex, place of residence (metropolitan, large urban area, medium sized urban area, small urban area, and rural area) and to compare cases and controls in terms of potential confounders. Conditional logistic regression was used to compute crude and adjusted odds ratios (OR) with 95% confidence intervals (CI). Regression models were built using the number of different dispensed medications as the main independent categorical variable. The reference group was set to one medication in order to define a basal risk since the vast majority of elderly people in Sweden consume at least one medication. The analyses were adjusted for marital status, CCI and number of FRIDs. We also stratified by dispensation of FRIDs during the last 30 days (yes/no).

Data management and statistical analyses were performed using SAS software version 9.2 (SAS Institute Inc., North Carolina).

### Ethical considerations

The participants did not provide their written or verbal informed consent to participate in the study. Rather, the need for written informed consent from the participants was waived by the Regional Ethical Review Committee in Stockholm when the project was reviewed as a whole.

According to Swedish regulation, register-based studies must be submitted for review and approval to regional ethical review boards. The application must specify among others why the researchers consider that informed consent is not necessary—or not feasible (e.g. in case of large studies as ours).

The study was approved by the Regional Ethical Review Committee in Stockholm, Sweden (2010/865-31/2 and 2011/15-32).

## Results

Most people who suffered from at least one fall injury during the study period (n = 64 399) were women (66%), aged between 80 to 89 years (52%) and not married (70%). The most common main diagnoses among the cases were femoral fracture (ICD10 S72; 39.7%), intracranial injury (S06; 9.6%), fracture of lumbar spine and pelvis (S32; 7.3%), and superficial injury of hip and thigh (S70; 5.6%). Table 1 illustrates that the fall injured, in general, consumed more FRIDs, had more hospitalisation days during the last year and that nearly 80% had a CCI score of 0, a proportion that is 10% lower than among controls. Married individuals had a lower risk of injurious falls compared to those from other categories of marital status. The risk of injurious falls increased in a dose-response manner with increasing number of FRIDs (Table 1), with similar associations among males and females (data not shown).

**Table 1:** Characteristics (%) of the Study Population, Stratified by Case and Control Status, n = 321 995.

Characteristics	Category	Total, %	Cases, %	Controls, %	Odds ratio^2^
		n = 321 995	n = 64 399	n = 257 596	OR (95% CI)
Marital status	Married	34.9	29.9	36.1	1 (REF)
	Unmarried	7.6	8.6	7.3	1.47 (1.42–1.52)
	Divorced	10.4	11.9	10.0	1.48 (1.43–1.52)
	Widowed	47.2	49.6	46.6	1.38 (1.35–1.41)
Number of FRIDs	0	59.5	42.0	63.9	1 (REF)
	1	14.8	18.1	14.0	2.00 (1.95–2.05)
	2	10.3	14.2	9.3	2.39 (2.32–2.46)
	3	6.9	10.4	6.1	2.72 (2.63–2.80)
	4	4.2	1.2	3.5	3.26 (3.14–3.39)
	≥5	4.3	8.1	3.3	3.96 (3.81–4.11)
Charlson comorbidity index groups the year prior to index date^1^	0	89.2	81.4	91.1	1 (REF)
	1–2	10.2	17.4	8.3	2.36 (2.30–2.42)
	3–4	0.4	0.8	0.3	2.76 (2.47–3.09)
	≥5	0.3	0.4	0.2	2.06 (1.78–2.39)
Number of hospitalisation days in the year prior to index date	0	76.2	64.1	79.2	1 (REF)
	1	2.6	3.2	2.5	1.63 (1.55–1.72)
	2–3	4.0	5.1	3.7	1.69 (1.60–1.79)
	4–7	5.1	7.2	4.6	1.95 (1.88–2.02)
	8–15	5.0	8.0	4.2	2.37 (2.89–2.45)
	15–30	4.4	7.6	3.6	2.63 (2.54–2.73)
	>30	2.7	4.8	2.2	2.79 (2.66–2.92)

^1^ Final grouping range from 0 (no relevant concomitant disease) to 9 (very relevant concomitant disease).

^2^ Matched for sex. age and area of residence.

**Fig 1:**
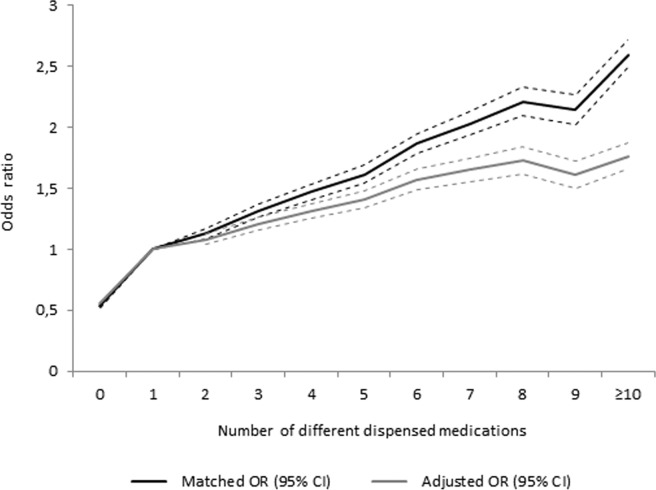
Odds ratio for injurious falls including 95% confidence intervals (dotted lines) by number of medications, matched and adjusted for marital status, Charlson Comorbidity Index, number of hospitalisation days and number of FRIDs.

All types of medications aggregated, number of medications was associated with an increased risk of fall injury (Table 2 and Fig. 1). The effect and trend observed in the matched analyses modified only marginally after adjusting for marital status, number of hospitalisation days and CCI, and just a minor downward adjustment was seen when FRIDs were taken into account. Using ten or more drugs was associated with an almost two-fold higher risk of hospitalization for fall injury (adjusted OR: 1.76, 95% CI: 1.66 to 1.88), after these factors were taken into account.

**Table 2:** Odds Ratio for Injurious Falls by Number of Medications. Adjusted for Marital Status. Charlson Comorbidity, Number of Hospitalisation Days and Number of Fall-Risk-Inducing Drugs (FRIDs).

Number of medications	Cases	Controls	Matched^1^	Adjusted for				
	n = 64 399	n = 257 596		Marital status	Charlson Comorbidity Index	Number of hospitalisation days	Number of FRIDs	All^2^
	%	%	OR (95% CI)	OR (95% CI)	OR (95% CI)		OR (95% CI)	OR (95% CI)
0	26.6	49.6	0.53 (0.52–0.55)	0.53 (0.51–0.55)	0.53 (0.51–0.55)	0.54 (0.52–0.56)	0.55 (0.53–0.57)	0.55 (0.54–0.57)
1	11.1	11.1	1 (REF)	1 (REF)	1 (REF)	1 (REF)	1 (REF)	1 (REF)
2	9.8	8.8	1.13 (1.09–1.17)	1.12 (1.08–1.17)	1.12 (1.08–1.16)	1.11 (1.07–1.15)	1.10 (1.06–1.15)	1.08 (1.04–1.12)
3	8.7	6.7	1.31 (1.26–1.37)	1.30 (1.25–1.36)	1.29 (1.24–1.34)	1.27 (1.22–1.32)	1.27 (1.21–1.32)	1.21 (1.16–1.26)
4	7.9	5.5	1.47 (1.41–1.53)	1.45 (1.39–1.51)	1.42 (1.36–1.48)	1.40 (1.34–1.46)	1.40 (1.34–1.47)	1.31 (1.25–1.37)
5	7.1	4.5	1.61 (1.55–1.69)	1.59 (1.52–1.66)	1.56 (1.49–1.63)	1.52 (1.45–1.59)	1.52 (1.45–1.60)	1.41 (1.34–1.48)
6	6.5	3.6	1.87 (1.78–1.95)	1.83 (1.75–1.92)	1.77 (1.69–1.86)	1.73 (1.65–1.81)	1.74 (1.65–1.83)	1.57 (1.49–1.66)
7	5.6	2.9	2.03 (1.93–2.13)	1.99 (1.90–2.09)	1.92 (1.83–2.01)	1.84 (1.75–1.93)	1.86 (1.76–1.97)	1.65 (1.56–1.75)
8	4.6	2.2	2.21 (2.10–2.33)	2.16 (2.05–2.28)	2.06 (1.96–2.17)	1.97 (1.87–2.08)	1.99 (1.87–2.11)	1.73 (1.62–1.84)
9	3.5	1.7	2.14 (2.02–2.27)	2.09 (1.97–2.21)	1.97 (1.86–2.09)	1.88 (1.77–1.99)	1.89 (1.77–2.03)	1.61 (1.50–1.72)
10+	8.7	3.5	2.59 (2.48–2.71)	2.52 (2.42–2.63)	2.30 (2.20–2.40)	2.15 (2.06–2.45)	2.19 (2.06–2.33)	1.76 (1.66–1.88)

^1^Matched for age. gender and area of residence.

^2^Adjusted for marital status. Charlson Comorbidity Index, number of hospitalisation days and number of FRIDs.

**Fig 2:**
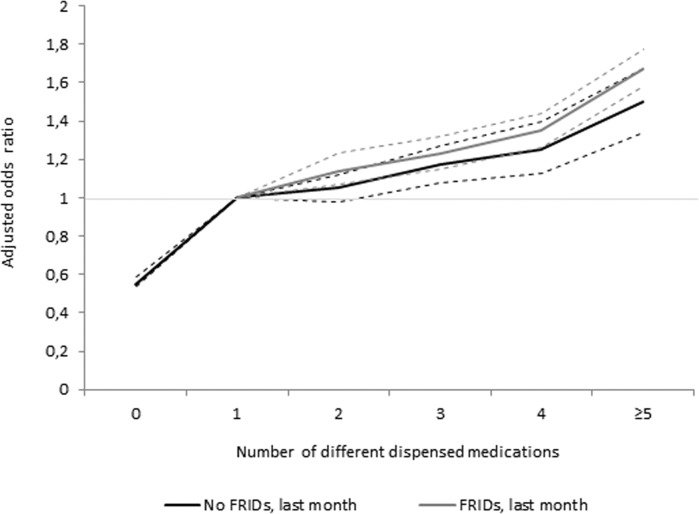
Odds ratio for injurious falls by number of medications stratified by dispensed FRIDs last 30 days, adjusted including 95% confidence intervals (dotted lines).

When stratified by use or non-use of at least one FRID during the last 30 days (Table 3 and Fig. 2), the association between number of medications and injurious falls was slightly weaker among those who had not used than among those who had (adjusted OR: 1.50, 95% CI: 1.34 to 1.67 vs. adjusted OR: 1.67, 95% CI: 1.58 to 1.77). There was a 10–20% higher increment in the risk for injurious falls with each additional medication among those persons using at least one FRID compared with those using none. Yet, the increased risk and trend remained when no FRIDs were dispensed, ranging from 5% with 2 medications (95% CI 0.98–1.12) to 50% with 5 or more medications (95% CI 1.34–1.67).

**Table 3:** Adjusted^1^ Odds Ratio for Injurious Falls by Number of Medications. Stratified by Dispensed FRIDs Last 30 Days.

Number of medications	No FRIDs	FRIDs
	(n = 191 557. 59.5%)	(n = 130 438. 40.5%)
	OR (95% CI)	OR (95% CI)
0	0.56 (0.54–0.59)	NA
1	1 (REF)	1 (REF)
2	1.05 (0.98–1.12)	1.14 (1.07–1.23)
3	1.17 (1.08–1.27)	1.23 (1.15–1.32)
4	1.25 (1.13–1.40)	1.35 (1.26–1.44)
5+	1.50 (1.34–1.67)	1.67 (1.58–1.77)

NA = All persons with at least one FRID will have at least one medication.

^1^Adjusted for marital status, Charlson Comorbidity Index, number of hospitalisation days.

## Discussion

### Main findings

Polypharmacy, defined as the use of five or more prescribed medications [26], is an acknowledged risk factor for fall injuries in different populations. Our study complements this by providing indication that the risk of injurious falls could occur even with less than five medications, indeed in some kind of graded manner with increasing number of prescribed medications. We also find that the progression in excess risk could be independent of the use of FRIDs and robust after adjustment for co-morbidity prior to the fall. To our knowledge, the latter findings have not been reported to date.

The risk difference between 0 and 1 medication in people with no FRIDs might be explained in two different manners that both find support in the literature. On one hand, it is plausible that the number of medication is merely a surrogate for health status; and number of medications is a valid proxy for overall comorbidity [27]. On the other hand, it is also possible that the consumption of medication, even for small numbers, engenders an increased risk of injurious falls. This has been observed even in robust patients with rather low levels of medication use [18].

Fall-risk scores currently used to determine the risk of falling in older adults [28], such as the Hendrich II model [29] and its adaptations [30, 31] take into account specific types of medications known to increase the risk of falls, but do not yet consider these in terms of the number of medications or polypharmacy which potentially could improve the sensitivity and specificity of such models. For prediction of clinical outcomes after fall injuries, where detailed information from medical records can be obtained, models taking into consideration number of medications has shown to be effective [32].

We also found, as expected, an increased risk of falls among those who had a high level of co-morbidity, the most important confounder of the association studied. We used the updated and validated weighted Charlson Comorbidity Index (CCI) to handle confounding by indication. Although the CCI does not cover the whole range of comorbidities, it is also used in other studies on falls among elderly. Additionally we used number of hospitalisation days as an alternative proxy for comorbidity. In this study, the effect of both measures of comorbidity was significant and tended to increase with the number of different dispensed medications in bivariate analyses. However, while the odds ratios were reduced in the adjusted model, the magnitude of the change was considerably lower than could have been expected. Apparently, the value of adjusting for comorbidity using the CCI is bigger when dealing with falls occurring among geriatric patients in hospital settings [33–35]. Therefore, validated or more sensitive comorbidity assessment instruments are needed for outcomes other than in-patient mortality, such as that of injurious falls occurring at community level.

To disentangle the role of disease or comorbidity and that of medication itself is studies like ours is challenging. In a recently study on a far smaller regionalpopulation-based sample we were able to control for a larger variety of (self-reported) health-related parameters. We observed that the strength of the association between number of medications and fall injuries leading to hospitalization was slightly—but not remarkably—reduced after adjusting for a range of significant risk factors characterizing individual demographics, lifestyle and health status [36]. That pharmacological interactions could come into play is plausible [37], i.e. due to apparently innocuous medications and the inappropriate use of medications, not detected using comorbidity assessments [38].

The idea of identifying fall risk inducing drugs (FRIDs) was first proposed in the early 1990s with a list that included mostly diuretics and psychotropics [39]. To date, there is still no consensus on which medications to include on the list and thus various different lists are used [5, 12, 40, 41]. Conducting our study in the Swedish context, we adhered to the list issued by the Swedish National Board of Health and Welfare [23], which is based on expert opinion and literature review. We observed that the effect of number of medications on the risk of injurious falls remained after adjusting for FRIDs and that the effect of the adjustment was more pronounced with an increasing number of medications. For two medications, for instance, the odds ratio changed from 1.13 to 1.08 whereas for 10 or more it went from 2.59 to 1.76. We assume that this can be attributable to the pharmacological interaction between medications and to the increased likelihood of inappropriate prescriptions—or combinations of medications—with increasing numbers.

We used individuals taking one medication as a reference group to define a basal risk since the vast majority of elderly people in Sweden consume at least one medication. For instance, the reported prevalence of ≥1 dispensed medications in 2006 almost reached 95% among individuals aged 80–89 years [42]. The highly protective adjusted ORs observed among those taking no medications in the various periods assessed prior to the index date depict these persons as a healthy group.

### Strengths and limitations

The strengths of this study not only relate to its national representativeness, large size and efficient design, but also to the high quality and coverage of the Swedish health registers. Also, the fact that we were able to confirm the dispensation of medications prior to the fall injury, thanks to the prescribed drug register, precludes the recall bias that characterizes conventional case-control studies. On the other hand, limitations include the use of dispensation as a surrogate for actual intake thus potentially leading to misclassification, particularly in shorter timeframes [43]; yet, there is nothing to suggest that this differs between cases and control. Further, the prescribed drug register does not include over-the-counter dispensed drugs or medications given during hospitalization, which may lead to an underestimation of drug use, albeit equally among cases and controls.

One possible reason for the observed effect relates to drug-drug interactions, which are likely to increase as more medications are combined. The reported prevalence of polypharmacy (≥5 medications) within a three-month period in Sweden was 39% among community-dwellers aged ≥65 years in 2008 [44], and the prevalence of potentially inappropriate drug use reached 12% [45]. Therefore, the large number of medications dispensed in Sweden, including those inappropriately prescribed, could be contributing to the occurrence of falls.

### Conclusion and implications

The use of medications needed to manage their multiple conditions combined with pharmacological factors (pharmacokinetics and pharmacodynamics) place the elderly at greater risk of drug-related side effects due to changes in body composition and impaired hepatic and renal functioning [5]. This, in turn, reemphasizes the need for health care professionals to pay attention to older people’s consumption of medication not only in kind but also in number. In older people, not only large but also small numbers of medications may affect the risk for them to sustain injurious falls. Although the mechanisms lying behind this are complex, the finding challenges the prevention strategies targeting either specific types of medications (FRIDs) or high numbers of them.
